# Perfectionism, obsessive–compulsive symptoms, and health-thinness-cleanliness motivations as primary correlates of orthorexia nervosa

**DOI:** 10.1007/s40519-026-01823-x

**Published:** 2026-03-14

**Authors:** Maddy Greville-Harris, Laura Vuillier, Catherine Talbot, Katherine Appleton

**Affiliations:** 1 Department of Psychology, Washington Singer Laboratories, Health and Life Sciences, University of Exeter, Exeter, EX4 4QG UK; 2 Department of Psychology Faculty of Science and Technology, Bournemouth University, Poole, BH12 5BB UK

**Keywords:** Orthorexia nervosa, Disordered eating, Dietary motivations, Perfectionism, Obsessive–compulsive symptoms

## Abstract

**Purpose:**

Orthorexia Nervosa (ON) is characterized by an obsessive preoccupation with eating foods perceived as healthy or clean. Despite growing research interest, risk factors and underlying motivations for ON remain poorly understood. This study examined demographic factors, psychological correlates, and dietary motivations associated with ON symptomatology, building on McComb and Mills' (2019) theoretical model.

**Methods:**

Participants (*N* = 697) completed validated measures of ON, alongside assessments of demographic factors (age, gender, socioeconomic status, dietary means), psychological variables (perfectionism, obsessive–compulsive symptoms, perceived control, perceived vulnerability to disease, past eating disorder diagnosis), and dietary motivations (thinness, health, cleanliness, muscularity, feeling mentally well).

**Results:**

Three dietary motivations: desires to be thin, healthy, and clean were significantly associated with ON symptoms across both measures. Perfectionism (specifically perfectionistic striving) and obsessive–compulsive symptoms (particularly the 'washing' subscale reflecting contamination concerns) emerged as the strongest psychological predictors of ON symptomatology. Demographic variables (age, gender, socioeconomic status) were not associated with ON symptoms, although lower perceived dietary means was associated with higher ON symptomatology. Results were largely replicated using both ON scales.

**Conclusion:**

ON appears driven by perfectionist striving and contamination-related obsessive–compulsive symptoms, alongside complex motivations that extend beyond health to also include thinness and cleanliness concerns. These findings suggest that ON represents a convergence of multiple motivational domains and highlights potential targets for intervention, including perfectionism-based and exposure–response prevention approaches adapted from obsessive–compulsive disorder and eating disorders treatments.

**Level of evidence:**

Level V, based on a descriptive study.

**Supplementary Information:**

The online version contains supplementary material available at 10.1007/s40519-026-01823-x.

## Introduction

Orthorexia Nervosa (ON) is a disordered eating style characterized by an obsessive preoccupation with eating foods perceived as healthy or "clean" [1, 2]. The focus in ON is food quality (rather than quantity), often driven by fears related to cleanliness and impurity of food [3, 4]. Although ON is not currently recognised in diagnostic manuals, it is associated with extreme control over food intake, rigid dietary rules [1], psychosocial impairment and distress [3, 5, 6]. Symptoms of ON include maladaptive beliefs about the pathogenic nature of ‘unhealthy’ foods, extreme guilt or anxiety at the prospect of dietary lapses, and debilitating compensatory behaviours [4, 5].

Despite growing awareness, ON seems to be frequently delegitimised or overlooked [7] likely due to the socially accepted nature of ‘healthy’ eating [8] and the absence of low body weight in ON presentations [9]. Although research on ON is expanding, little attention has been given to underlying risk factors and motivational drivers in ON. McComb and Mills [10] identified several potential psychological risk factors for ON, including perfectionism, obsessive–compulsive (OC) symptoms, fear of losing control, and perceived vulnerability to illness. However existing findings are constrained by: (1) the use of ON measures with poor psychometric validity [11], (2) limited multi-factor models, and: (3) insufficient exploration of the motivations behind dietary restriction in ON. This study aims to address the aforementioned limitations.

### Demographic factors and ON

McComb and Mills [10] highlight the potential role of several demographic factors in the aetiology of ON. The role of gender and age are well-recognised in disordered eating [12, 13]. However, in ON, some studies report higher ON symptomatology in females [14], others in males [15], and several studies find no gender differences [16, 17]. A recent study found non-binary individuals report lower ON symptomology than cisgender individuals, possibly due to less pressure to conform to gender norms [18]. Research on age and ON is sparce, although adolescence has been suggested as a time of increased ON symptomatology [19].

Similarly, there is limited research exploring socio-economic status (SES) as a risk factor for ON, although some research suggests higher SES is linked to increased ON symptomatology [20]. Given that ON has been linked to increased consumption of organic, artisanal, or sustainable foods [21], having the means to access preferred foods may also be an important factor.

### Perfectionism, perceived control and OC symptomatology

Among the psychological correlates for ON, perfectionism is perhaps the most extensively examined within existing literature. Perfectionism is a well-established transdiagnostic risk factor for disordered eating [22]. However, while in many eating disorders (EDs) perfectionism seems to target pursuit of the ‘perfect’ weight or shape, ON is postulated to focus more on the pursuit of perfect ‘health’ [23]. A recent meta-analysis found a consistent link between ON and perfectionism, regardless of the measurement tools used [23]. Specifically, perfectionism striving (setting unrealistically high expectations) was more strongly linked to ON than perfectionist evaluative concern (highly critical self-evaluation, concern of evaluation from others), suggesting that ON may be more strongly associated with high standards for the ‘perfect’ diet, rather than by concern over external evaluation or judgement.

Given that the aetiology of ON also includes an obsessive fixation on diet and healthy eating [1], OC symptoms are also implicated in ON [10]. OC symptoms comprise the key facets of obsessions (intrusive/repeated cognitions about a topic), contamination fears (related to washing compulsions), checking (checking objects/places) and ordering (having to do/have things in a certain order) [24]. A meta-analysis indicated a small but significant association between ON symptomatology and OC symptoms [25]. However, like much ON research, studies were limited by the use of ON measures (such as the ORTO-15 [26]) with low psychometric properties [11], as well as a lack of focus on the separate OC facets.

McComb and Mills [10] also tentatively suggest the role of perceived control in ON, specifically fear of losing control. This possible correlate has been largely overlooked in the context of ON, although qualitative research has highlighted experiences of a punishing drive for control [8], with participants with ON symptomatology describing the way(s) in which healthy eating allows them to take control of their diet and other aspects of their lives [5, 8]. An association between ON and high beliefs around health controllability has also been identified [5], with evidence that ON behaviours may be used to cope with the perceived uncontrollability of emotions [27]. However, to our knowledge no quantitative research to date has explicitly looked at the potential role of sense of control in ON.

### Perceived vulnerability to disease

As suggested by McComb and Mills [10] negative emotional states, specifically health anxiety, may also be linked to higher ON symptomatology. A small number of studies indicate positive associations between health anxiety and ON symptomatology [5, 28, 29] although McComb and Mills [10] postulate that it is perceived vulnerability to disease (PVD) (the perceived susceptibility to becoming ill from infectious diseases [30]—a facet of health anxiety) that is particularly important in the aetiology of ON. Qualitative research highlights that individuals with ON symptomatology report avoiding ‘unhealthy’ foods to avoid chemicals, toxins, and preventable illnesses [5] and thus concerns about illness may be a precursor to ON food restriction.

### Past ED history

McComb and Mills (2019) also highlight several factors related to ED symptomatology which may be implicated in ON, including: (1) restrictive eating, (2) current ED and: (3) past ED. This is also highlighted in the recent consensus paper by Donini et al. [2] which suggests that ON may be associated with the migration to or from other forms of EDs. For example, Donini et al. [2] suggest that ON may be a more socially acceptable form of restriction for individuals with or in recovery from AN. Given that a risk factor, by definition, must theoretically be independent of current pathology [31] *current* eating symptomatology does not fit this premise. However, history of past ED (without a current diagnosis) may be a valuable correlate, as ON behaviours may emerge as replacements of previous restrictive eating patterns [32], suggesting ON could serve as a coping mechanism post-ED recovery.

### Motivation for an ‘ON’ diet

While there is a growing list of potential psychological correlates for ON, the motivation behind ON symptomology has largely been overlooked. ON is traditionally distinguished from Anorexia Nervosa (AN) by its focus on health rather than weight ideals [2, 4]. However, the focus on being ‘healthy’ or ‘clean’ in ON is perhaps more complex. In a study on underlying motivations [33], the main dietary motivation for ON was weight control, with sensorial appeal and emotion regulation also playing a role. Recent qualitative research in ON also suggests that weight and health are conceptualised as intertwined, such that ‘health’ was conceptualised as incorporating body ideals around fitness and tone (although not thinness per se) as well as the pursuit of a ‘healthy mind’ and body[5]. Moreover, for some individuals, ON may be a socially acceptable form of food restriction, characterised by issues related to body shape and body fat [3]. Thus, given the complexities and multifaceted nature of possible motivations for healthy diets in ON, it is important to explore such motivations for ON in more detail.

### Study aims

Despite increasing interest in ON research, the risk factors and underlying motivations for ON remain poorly understood. This study therefore builds on McComb and Mills’ [10] model, and aims to explore the relative importance of demographic factors (gender, age, SES, and dietary means), potential psychological correlates (perfectionism, OC symptoms, perceived control, PVD and past ED diagnosis) and dietary motivations (to be thin, toned, muscular, healthy, clean, and mental wellness) in explaining symptomatology in ON. This is an exploratory study which will investigate: 1) which of the above factors are significant correlates of ON symptomatology within a multiple regression model, and: 2) which subscales of significant primary correlates are associated with ON symptomatology.

## Methods

### Participants

In total, 697 participants took part in the study^1^ recruited from Bournemouth University (*n* = 435) and the community (*n* = 262). The study was made available to psychology students at Bournemouth University via an online university-based research participant pool (Sona Systems ^®^). Students who took part received course credits for participation. Recruitment also took place via advertising in the local area (e.g. health food shops), and via advertisement on social media.

Footnote 1: A subset of this dataset (*n* = 507) was used in an earlier study: Greville-Harris M, Vuillier L, Goodall S, et al., (2024) Striving for the perfect diet? The mediating role of perfectionism in the relationship between obsessive compulsive symptoms and traits of Orthorexia Nervosa. *J Eat Disord*. The current dataset includes additional data collected since that publication, and focuses on a wider range of factors.

## Materials

### Demographics

Gender, age, student status, ethnicity and ED diagnoses self-report data were collected. Participants were asked to specify (yes/no) whether they: (1) ever had a diagnosis of an ED; (2) had a current diagnosis of an ED, and: (3) self-identified with the label of Orthorexia Nervosa.

Subjective SES was measured using the MacArthur Ladder [34]; a widely used measure in health and wellbeing research [35]. Participants were given the following description: *‘The ladder represents where people stand in society. At the top of the ladder are the people who are the best off, those who have the most money, most education, and best jobs. At the bottom are the people who are the worst off, those who have the least money, least education, worst job, or no job.’* Participants were asked to rate their perceived position on the ladder scored from ‘*1- worst off*’ to ‘*10- best off*’. Dietary means was then measured using one item ‘*I have the means to eat the foods that I want*’ rated on a sliding scale from ‘*1-totally disagree*’ to ‘*10-totally agree*’. This item was included to specifically measure perceived means to access desired foods, alongside the general SES measure.

### The Dusseldorf Orthorexia Scale—English Version

The Dusseldorf Orthorexie Scale English version- E-DOS [36] is a psychometrically validated questionnaire assessing ON-related eating thoughts and behaviours [11], comprising ten items rated on a 4-point Likert scale from ‘*1—this does not apply to me*’ to ‘*4—this applies to me*’. Total scores range from 10 to 40, where higher scores indicate greater ON symptomatology. A preliminary cut-off of ≥ 30 is used to indicate likely ON (4.30% of our sample), with a score ≥ 25–29 suggesting risk of ON (9.47% of our sample) [36]. This measure had good internal consistency in our sample (α = 0.85).

### The Eating Habits Questionnaire

Given that there is no gold standard measurement tool for ON [11], the Eating Habits Questionnaire- EHQ [37] was used as a confirmatory measure of ON symptomatology. This is a 21-item measure of ON-related cognitions, behaviours and emotions rated on a 4-point Likert scale from ‘*1-false, not at all true*', to ‘*4- very true*’, with higher scores indicative of likely difficulties in this area (score range 21–84). This scale had excellent internal consistency in our sample (α = 0.91).

### Perfectionism

Perfectionism was assessed using the validated Frost Multidimensional Perfectionism Scale- brief [38] which measures two facets of perfectionism: (1) achievement striving and (2) evaluative concern. The scale comprises 8 items (4 for each subscale) on a 5-point scale from ‘*1—strongly disagree*’ to ‘*5—strongly agree*’. Total scores ranged from 8–40 (subscale range from 4–20), with higher scores denoting higher perfectionism. This scale had good internal consistency in our sample (0.87) with good subscale reliability scores (α = 0.84–0.87).

### OC symptomology

OC Symptomology was measured using The Obsessive–Compulsive Inventory—Revised (OCI-12) [39], a psychometrically validated 12-item questionnaire assessing OC symptoms over the past month. Items are responded on a 5-point scale, scored from ‘*0—not at all*’ to ‘*4—extremely*’, and summed to provide a total score. Scores range from 0—48 (0–12 for subscales), with higher scores indicating greater levels of symptomatology. The OCI-12 has four subscales (1) washing, (2) checking (3) ordering and: (4) obsessing. This scale had excellent internal consistency in our sample (α = 0.91) with good reliability for subscales (α = 0.77–0.89).

### Sense of control

Sense of control over one’s life was measured using the Sense of Control Scales from the Midlife Development Inventory (MIDI) [40]. This questionnaire uses 12 items to measure: 1) personal mastery—what people feel they can control themselves (4 items), and: 2) personal constraints—what people feel they cannot control (8 items). Items are rated on a 7-point scale from ‘*1*—*strongly disagree*’ to ‘*7*—*strongly agree*’, with higher scores indicating greater perceived control (score range 12–84). This scale had good internal consistency in our sample (α = 0.87) with good reliability for subscales (α = 0.78–0.86).

### Perceived vulnerability to disease (PVD)

The PVD questionnaire [41] uses 15 items to assess two factors: 1) perceived infectability—beliefs about personal susceptibility to infectious diseases (7 items) and: 2) germ aversion—the emotional discomfort associated with potential contagion from an infectious disease (8 items). A 7-point Likert scale is used, scored from ‘*1*—*strongly disagree*’ to ‘*7*—*strongly agree*’, with higher scores indicating greater levels of perceived vulnerability (score range 8–48). This scale had good internal consistency in our sample (α = 0.81) with good reliability for subscales (α = 0.73–0.88).

### Dietary motivations

Participants were asked to rate their agreement with the following six statements rated from ‘*1- totally disagree*’ to ‘*10- totally agree*’: ‘*I eat the food that I do to….’ (1) ‘be thin’, (2) ‘be toned’, (3) ‘be muscular’, (4) ‘be healthy’, (5) ‘be clean’, (6) ‘feel good mentally’*. Given the lack of a concise validated measure of dietary motivations in ON, we created these items based on existing literature on potential motivational domains in ON [5, 33].

### Procedure

The composite online questionnaire was hosted on Qualtrics XM and included all study information and consent procedures. The study took 20–30 min to complete and was approved by the Research Ethics Committee of Bournemouth University (ID 39332).

### Statistical analysis plan

We ran a hierarchical regression (Model 1) with demographic variables (gender, age, SES, dietary means) entered in Block 1, and psychological factors (perfectionism, OC-symptoms, sense of control, PVD and past ED) in Block 2. Food motivations (to be thin, toned, muscular, healthy, clean, mentally well) were entered into Block 3. This allowed us to explore overall correlates of ON, as well as whether: (1) demographic factors were associated with ON, (2) psychological correlates helped to explain symptoms of ON after accounting for demographic factors, and: (3) motivational factors showed predictive power beyond the aforementioned potential correlates. This model used the total scale scores for predictors- subscales were not included to avoid multicollinearity and suppression effects. Given the number of predictors in model 1 (15), Bonferroni correction was applied to give a more conservative cut-off for significance (*p* = 0.003).

In Model 2, subscales for any significant predictors from Model 1 were explored, to ascertain which subcomponents of the psychological factors were potentially important in explaining symptoms of ON. As in Model 1, a multiple linear regression was run using the Enter method. Given that Model 2 focused on subscales (rather than the relative contribution of multiple demographic, motivational and psychological correlates) all predictors were entered in one block. For model 2 (10 predictors) Bonferroni correction was applied to give a more conservative cut-off for significance (*p* = 0.005).

Models were run with all available data; number of participants varied in each model as a result of missing data for specific questions, questionnaire items. Assumption checks were carried out for all models (see Supplementary Materials 1).

The above models were run with EDOS as the outcome variable (Models 1 and 2) and re-run using EHQ as a confirmatory analysis (Models S1 and S2 in Supplementary Materials 2).

## Results

### Respondents

Demographic data are outlined in Table 1, including scores for all variable scales and subscales for the full sample.

**Table 1:** Demographic and descriptive variable data (*N* = 697)

Demographic, motivational and psychological variables	Mean (SD)		Range min–max
Age	28.12 (13.36)		16–86
SES	5.93 (1.36)		1–10
Dietary means	7.05 (2.27)		1–10
ON symptomatology (E-DOS)	17.73 (5.82)		10–40
ON symptomatology (EHQ)	35.96 (10.09)		21–81
Perfectionism- sum score	23.04 (7.07)		8–40
Achievement striving	12.22 (4.03)		4–20
Evaluative concerns	10.82 (4.12)		4–20
OC symptom sum score	14.19 (10.27)		0–48
Checking	3.41 (2.95)		0–12
Ordering	4.02 (3.22)		0–12
Washing	2.20 (2.86)		0–12
Obsessing	4.57 (3.63)		0–12
PVD sum score	56.17 (14.29)		16–105
Perceived infectability	25.87 (9.41)		7–49
Germ aversion	30.30 (8.94)		9–56
Sense of control (MIDI) sum score	56.33 (12.31)		18–84
Personal mastery	20.63 (4.15)		4–28
Personal constraints	35.70 (9.78)		11–56
Motive for diet items…			
To be thin	4.32 (2.55)		1–10
To be toned	4.00 (2.58)		1–10
To be muscular	3.53 (2.48)		1–10
To be healthy	6.42 (2.50)		1–10
To be clean	4.69 (2.71)		1–10
To feel good mentally	6.51 (2.53)		1–10
	Count (%)		
Gender			
Male	77 (11.05)		
Female	607 (87.09)		
Non binary/other gender classification	10 (1.43)		
Ever had an ED (Yes)	50 (7.17)		
Currently have an ED (Yes)	12 (1.72)		
Meet EDOS criteria for ON risk (Score ≥ 25–29)	66 (9.47)		
Meet EDOS criteria for likely ON (Score ≥ 30)	30 (4.30)		

### Demographic, motivational and psychological correlates associated with ON symptomatology- E-DOS

#### Model 1

Models for block 1 and then each added block were significant (see Table 2), with the final model explaining 38.5% of the variance in ON symptomatology scores.

**Table 2:** Significance values and variance explained by each Block for Model 1

	F (df)	*p*	*R*^2^	*R*^2^ adjusted
Block 1	3.16 (5, 671)	.008	.023	.016
Block 2	13.72 (10, 666)	< .001	.171	.158
Block 3	25.81 (16, 660)	< .001	.385	.370

Block 1 = demographic variables; Block 2 = Block 1 + psychological correlates; Block 3 = Block 2 + dietary motivations

After applying Bonferroni corrections, (1) lower perceived dietary means, and higher: (2) perfectionism, (3) OC symptoms, as well as motivations: (4) to be thin, (5) to be healthy and: (6) to be clean, were significantly associated with higher levels of ON symptomatology (see Table 3). No other predictors were significant within Model 1.

**Table 3:** Coefficients for Model 1 (*N* = 677)

		Block 1					Block 2					Block 3			
Variable	Β		SE	*β*	*p*	Β		SE	*β*	*p*	Β		SE	*β*	*p*
Gender															
* Male–Female*^*△*^	−0.79		0.71	−		−0.71		0.67	−0.04		−0.02		0.60	−.001	
* NB-Female*^*△*^	−1.41		1.86	−0.03		−2.97		1.73	−0.06		−1.08		1.50	−0.02	
* Male-NB*^*□*^	−0.62		1.95	−0.01		−2.25		1.82	−0.05		−1.06		1.58	−0.02	
Age	−0.02		0.02	−0.03		0.02		0.02	0.05		0.02		0.02	0.05	
SES	0.17		0.18	0.04		0.24		0.17	0.06		0.06		0.15	0.01	
Dietary Means	−0.39		0.11	−0.15	**	−0.33		0.10	−0.13	**	−0.28		−0.09	−0.11	**
Perfectionism						0.26		0.03	0.32	**	0.17		0.03	0.21	**
OC Symptoms						0.11		0.03	0.19	**	0.09		0.02	0.16	**
Perceived Control						0.04		0.02	0.07		.001		0.02	.001	
PVD						−0.02		0.02	−0.04		−.005		0.02	−0.01	
Past ED						0.30		0.93	0.01		0.88		0.81	0.03	
To be thin											0.68		0.09	0.30	**
To be toned											−0.11		0.11	−0.05	
To be healthy											0.41		0.12	0.18	**
To be muscular											0.16		0.11	0.07	
To be clean											0.29		0.09	0.14	**
To feel good mentally											0.03		0.10	0.02	

^△^Female as reference category; ^*□*^Nonbinary (NB) as reference category; ** = *p* ≤ .001; * = *p* ≤ .003

#### Model 2

A multiple linear regression (Model 2) was then run including subscales for psychological predictors that were significant in Model 1 (perfectionism and OC symptoms) alongside all other significant individual predictors.

Model 2 was significant (*F*(10, 673) = 42.14, *p* < 0.001) and explained 38.5% of the variance in ON symptomatology (*R*^2^ = 0.385, *R*^2^ adj = 0.376). After applying Bonferroni corrections, lower dietary means, higher perfectionistic striving, higher OC washing, and higher motivations to be thin, clean and healthy were significant predictors of increased ON symptomatology (see Table 4).

**Table 4:** Coefficients for Model 2 (*N* = 684)

Variable	Β	SE	*β*	*p*
Dietary means	−0.28	0.08	−0.11	**
Perfectionism				
EC	0.11	0.06	0.08	
Striving	0.24	0.05	0.17	**
OC symptoms				
Washing	0.24	0.08	0.12	*
Checking	−0.04	0.08	−0.02	
Ordering	0.04	0.07	0.02	
Obsessing	0.08	0.07	0.05	
Dietary motivation				
To be thin	0.66	0.08	0.29	**
To be clean	0.28	0.08	0.13	**
To be healthy	0.46	0.09	0.20	**

^**^ = *p* ≤ .001; * = *p* ≤ .005

See Supplementary Materials 2 for secondary models run with the EHQ instead of the EDOS. All predictors reaching significance remained the same for the EHQ models, except for dietary means (no longer significant, *β* = −0.09) and age (reaching significance, *β* = 0.11, *p* < 0.001).

## Discussion

This study aimed to test a comprehensive multifactorial model to explore demographic, psychological and motivational correlates associated with ON. Building on McComb and Mills’ model [10], we examined whether demographic factors (age, gender, SES, dietary means), psychological correlates (perfectionism, OC symptoms, perceived control, past ED) and dietary motivations (to be thin, toned, healthy, muscular, clean or to feel mentally well) were significantly associated with ON symptoms. Our findings indicated that dietary motivations related to thinness, health, and cleanliness, as well as perfectionism, OC symptoms, and perceived dietary means were significant correlates of ON symptomatology.

### Dietary motives for thinness, healthiness and cleanliness in ON

Three of the self-reported dietary motivations—the desire to be thin, healthy and clean—were significantly associated with ON symptomatology. Interestingly, motivation for thinness was most strongly associated with ON symptomatology. Health was also a significant motivator associated with ON, as well as motivations for cleanliness (albeit less strongly). These finding contrast with the proposed criterion in the ON consensus paper [2]. Although current expert consensus [2] acknowledges that, in Western countries, health ideals often overlap with thinness ideals, it posits health as the primary driver of ON. Our findings highlight the complexity of ON aetiology and measurement, and are in line with previous work suggesting that motivations in ON mainly focus on weight control [33]. This further illustrates the complex relationship between health and weight motives in ON [5, 42] as well as the potential for ON to allow a socially acceptable form of food restriction [2, 3].

### Psychological correlates (perfectionism, OC symptoms) and ON symptomatology

While the motivational factors were most strongly associated with ON, our models also consistently found that perfectionism (specifically perfectionistic striving) and, to a lesser extent, OC symptoms (specifically ‘washing’ due to fear of contamination) were associated with ON symptomatology. These findings are consistent with previous literature linking orthorexic eating patterns with perfectionism [43], and aligns with recent work [23], which suggests that perfectionism striving is more strongly linked to ON than evaluative concern. This indicates that the focus in ON tends to lie on internal standards of health and cleanliness, rather than external validation in terms of expectations towards, or from, others [44].

Similarly, our OC symptomatology findings support research suggesting a small, but significant association, between ON symptomatology and OC symptoms [25]. However, our finding that the ‘washing’ subscale—which comprises contamination fears and washing compulsions—was predictive of ON is novel. This may reflect underlying beliefs around personal responsibility for avoiding illness and staying well in ON, outlined in previous qualitative research [5]. Healthism discourses in ON which construe physical and mental health as an individual’s personal responsibility [45] may also explain this link between contamination fears, washing behaviours, and the need for clean and healthy diets; those with ON have reported feeling responsible for avoiding preventable illness through healthy eating, avoiding toxins and harmful chemicals [5, 8]. Avoiding contamination through washing behaviours may serve a similar purpose in those with ON tendencies.

### Perceived dietary means

Interestingly, lower perceived dietary means was significantly associated with ON, in models using the E-DOS measure for ON, though this association did not reach significance in models using the EHQ. While this may indicate low dietary SES as an important correlate, an alternative explanation is that individuals with stronger ON tendencies view their dietary ideals—often associated with costly food practices [21]—as financially unattainable. It may not be the case that limited resources contribute to ON, but rather that those pursuing ON tendencies *feel* constrained by their resources in striving for a ‘perfect,’ and often expensive, diet.

### Other potential correlates of ON

Unlike in previous studies (e.g. [5, 28, 29, 32]), we did not find significant associations between ON and PVD, perceived control, past ED diagnosis or motivations to be toned, muscular or mentally well. Moreover, in our sample age, gender and SES were not associated with ON using the E-DOS. These findings may be specific to our sample, or may also demonstrate more clearly the relative importance of the predictors that were found to be significantly associated with ON when considering a number of potentially predictive factors together. This relative importance is clearly seen in our analyses, highlighting the value of our approach of considering a range of potential correlates in a single model. However, as with all regression models, it is important to note that a lack of statistical significance for predictors—when considered alongside other stronger predictors—does not imply the absence of any relationship with ON per se.

## Implications and future research

The present findings suggest that the motivational drivers for ON are multifaceted, encompassing not only a desire for health and cleanliness but also a strong pursuit of thinness. Given ON’s perfectionistic emphasis on internal striving, it may be fruitful for future research to explore whether the motivation for thinness in ON is more rooted in intrinsic motivations—such as self-optimization or purity—rather than in external evaluation of body image. If so, this would align with early ON research [46] and could represent a key distinction between ON and other EDs such as AN, where body image disturbances are strongly implicated [47].

Our results also suggest that both striving and contamination-related OC- symptoms may play a role in the aetiology of ON and could serve as valuable targets for intervention. Specifically, striving—characterised by setting unrealistic high expectations—may contribute to the rigid health rules observed in ON. This could be addressed through adapted perfectionism-based interventions used in other EDs (e.g. [48, 49]). Additionally, the role of the ‘washing’ sub facet of OC highlights the importance of contamination concerns in ON, which may reinforce restrictive eating behaviours through fears of impurity or disease [8]. This also presents a possible mechanism of action to focus on in ON treatment, and highlights the potential for adapting evidence-based approaches for OCD such as exposure and response prevention strategies [50]

Finally, although we observed some demographic associations with ON (e.g., age, dietary means), further research is needed in more diverse samples to understand how factors such as gender, cultural background, and SES influence the aetiology of ON symptomatology.

## Strengths and limitations

This study is novel in exploring a comprehensive multifactorial model of potential demographic, psychological and motivational factors associated with ON, and in using two psychometrically valid measured of ON. However, this work is subject to several limitations. First, the cross-sectional design precludes conclusions about causality. While we identified associations between certain correlates and ON symptomatology, longitudinal data are needed to determine whether these factors predict the onset/escalation of ON over time. Second, a subset of the data used in this study (*n* = 507) was previously included in a published paper examining the relationship between perfectionism and ON using a mediation model [51]. However, the current analysis adopts a very different approach: examining multiple potential correlates in a multifactorial framework, rather than only focusing on perfectionism and OC symptoms. As such, the current paper suggests that perfectionism and OC symptoms play an important role in ON above and beyond many other risks factors, although we recommend that research using an independent sample confirm our findings. Third, dietary motivations were assessed using single-item questions. Although this allowed for broad coverage of different motives, the lack of multi-item scales limits the depth and reliability of these measures and does not consider how combinations of motives (e.g., thinness and health) may interact. Fourth, a large proportion of the study participants (62.4%) were students. While we targeted students due to the high prevalence of eating disorder symptomatology reported by this population [52], this limits the generalisability of our findings to other populations. Finally, while we focus on perfectionism, other personality dimensions such as neuroticism—highlighted in previous research (e.g., [10])—were omitted here and warrant exploration in future models.

### What is already known

Orthorexia Nervosa (ON) is characterised by obsessive preoccupation with eating foods perceived as healthy, but potential risk factors and motivational drivers remain poorly understood. Previous research has suggested potential links between ON and factors such as perfectionism, but multifactorial models of potential correlates of ON have not been implemented, and findings have been limited by poor measurement tools.

### What this study adds

This study explores multiple potential risk and motivational factors implicated in ON using a multifactorial model. It demonstrates that ON is associated with motivations that extend beyond health to include desires for thinness and cleanliness. Perfectionist striving, contamination-related OC symptoms (specifically washing compulsions) and lower perceived dietary means were also associated with higher ON symptomatology. These findings highlight potential intervention targets, including perfectionism-based treatments and exposure–response prevention strategies adapted from obsessive–compulsive disorder approaches.

## Supplementary Information

Below is the link to the electronic supplementary material.

Supplementary Material 1.

## Data Availability

The datasets generated during and/or analysed during the current study are available from the corresponding author on reasonable request.
