# Supplementary material for: Perfectionism, obsessive–compulsive symptoms, and health-thinness-cleanliness motivations as primary correlates of orthorexia nervosa
Source: Eat Weight Disord. 2026 Mar 14;31(1):49. doi: 10.1007/s40519-026-01823-x (PMC13219126; doi:10.1007/s40519-026-01823-x)
Supplement: Supplementary file 1 — Supplementary Material 1. [file 40519_2026_1823_MOESM1_ESM.docx]

**Supplementary Materials 1. Assumption Checks for Regression Analyses**

Assumption checks were carried out for each regression model for the main analyses and supplementary analyses. Durbin Watson statistics ranged from 1.82-2.03 for all regression models indicating no issues with autocorrelation. VIF was well below 10 for all models with tolerance scores above 0.2 indicating no issues with multicollinearity. Residuals were also normally distributed for all models.

There were several outliers with casewise diagnostics with SD greater than 3 (Model 1, n=5; Model 2, n=3; Model S1, n=7; Model S2, n=7). However removing these outliers did not qualitatively alter the results, so all outliers were included in the final models reported here. There were also cases with high leverage values (Model 1, n=14; Model 2, n=0; Model S1, n=15; Model S2, n=0). Again, removing these cases did not alter the results, and so all cases were included in the final models.

**Supplementary Materials 2: Potential Risk Factors Associated with ON symptomatology- Regression Models using the EHQ as the measure of ON.**

Model S1

The same process from the main analyses was then re-run with EHQ as the measure of ON symptomatology. We ran a hierarchical multiple regression model with demographic variables entered in Block 1, psychological risk factors entered in Block 2, and dietary motivations entered in Block 3. Models for all blocks were significant (see Table S1), with the final Block explaining 39.1% of the variance in ON symptomatology scores.

Table S1. Significance values and variance explained by each Block for Model S1.

|  | F (df) | *p* | R^2^ | R^2^ adjusted |
| --- | --- | --- | --- | --- |
| Block 1 | 2.87 (5,670) | .014 | .021 | .014 |
| Block 2 | 14.76 (10,665) | <.001 | .182 | .169 |
| Block 3 | 26.43 (16,659) | <.001 | .391 | .376 |

After applying Bonferroni corrections, higher levels of perfectionism, OC traits, as well as motivation to be thin, to be healthy and to be clean, were all significantly associated with higher levels of ON symptomatology when using the EHQ measure. However age was also a significant predictor in Block 3, with higher age predicting higher levels of ON. Moreover lower dietary means was only a significant predictor of ON in Block 1, and higher perceived control was a significant predictor of ON symptomatology but only in Block 2 (See Table S2).

Table S2. Coefficients for Model S1.

|  | | Block 1 | | | | | Block 2 | | | | | Block 3 | | | |
| --- | --- | --- | --- | --- | --- | --- | --- | --- | --- | --- | --- | --- | --- | --- | --- |
| Variable | Β | | SE | *β* | *p* | Β | | SE | *β* | *p* | Β | | SE | *β* | *p* |
| Gender |  | |  |  |  |  | |  |  |  |  | |  |  |  |
| *Male-Female^△^* | -1.54 | | 1.23 | -0.05 |  | -1.36 | | 1.14 | -0.04 |  | -1.09 | | 1.03 | -0.03 |  |
| *NB-Female^△^* | -3.76 | | 3.20 | -0.05 |  | -6.60 | | 2.96 | -0.08 |  | -3.57 | | 2.57 | -0.04 |  |
| *Male-NB^□^* | -2.21 | | 3.37 | -0.02 |  | -5.24 | | 3.11 | -0.06 |  | -2.48 | | 2.71 | -0.03 |  |
| Age | 0.01 | | 0.03 | 0.02 |  | 0.08 | | 0.03 | 0.11 |  | 0.09 | | 0.03 | 0.12 | ** |
| SES | 0.58 | | 0.31 | 0.08 |  | 0.59 | | 0.30 | 0.08 |  | 0.21 | | 0.26 | 0.03 |  |
| Dietary Means | -0.55 | | 0.18 | -0.12 | * | -0.47 | | 0.17 | -0.11 |  | -0.38 | | 0.15 | -0.09 |  |
| Perfectionism |  | |  |  |  | 0.44 | | 0.06 | 0.31 | ** | 0.29 | | 0.05 | 0.20 | ** |
| OC Traits |  | |  |  |  | 0.22 | | 0.05 | 0.22 | ** | 0.19 | | 0.04 | 0.19 | ** |
| Perceived Control |  | |  |  |  | 0.12 | | 0.03 | 0.15 | ** | 0.04 | | 0.03 | 0.05 |  |
| PVD |  | |  |  |  | 0.01 | | 0.03 | 0.01 |  | 0.03 | | 0.03 | 0.05 |  |
| Past ED |  | |  |  |  | -0.52 | | 1.59 | -0.01 |  | 0.93 | | 1.39 | 0.02 |  |
| To be thin |  | |  |  |  |  | |  |  |  | 0.68 | | 0.15 | 0.17 | ** |
| To be toned |  | |  |  |  |  | |  |  |  | 0.19 | | 0.20 | 0.05 |  |
| To be healthy |  | |  |  |  |  | |  |  |  | 0.68 | | 0.20 | 0.17 | ** |
| To be muscular |  | |  |  |  |  | |  |  |  | 0.41 | | 0.18 | 0.10 |  |
| To be clean |  | |  |  |  |  | |  |  |  | 0.48 | | 0.15 | 0.13 | * |
| To be feel good mentally |  | |  |  |  |  | |  |  |  | 0.18 | | 0.17 | 0.05 |  |

Note: ^△^Female as reference category; *^□^*Nonbinary (NB) as reference category; **= *p*≤.001; * = *p*≤.003˙

Model S2

A multiple linear regression (Model S2) was then run including only subscales for psychological predictors that were significant in Model S1 in the final block (perfectionism, and OC traits) alongside demographic factors (age) and dietary motivation items (to be thin, clean, healthy). All variables were entered using the ENTER method in one block.

Model S2 was significant (*F*(10, 672) = 41.32, *p*<.001) and explained 38.1% of the variance in ON symptomatology (R^2^=.381, R^2^ adj=.372). After applying Bonferroni corrections, higher age, higher perfectionistic striving, higher OC washing, and higher motivations to be thin, clean and healthy were significant predictors of increased ON symptomatology (see Table S3).

Table S3. Coefficients for Model S2.

| Variable | Β | SE | *β* | *p* |
| --- | --- | --- | --- | --- |
| Age | 0.08 | 0.03 | 0.11 | ** |
| Perfectionism |  |  |  |  |
| EC | -.006 | 0.10 | -.002 |  |
| Striving | 0.58 | 0.09 | 0.23 | ** |
| OC Traits |  |  |  |  |
| Washing | 0.48 | 0.14 | 0.14 | ** |
| Checking | 0.16 | 0.14 | 0.05 |  |
| Ordering | 0.11 | 0.13 | 0.04 |  |
| Obsessing | 0.09 | 0.12 | 0.03 |  |
| Dietary Motivation |  |  |  |  |
| To be thin | 0.86 | 0.13 | 0.22 | ** |
| To be clean | 0.59 | 0.15 | 0.16 | ** |
| To be healthy | 0.83 | 0.16 | 0.21 | ** |

Note: **= *p*≤.001
